# Effect of Axial Eye Length on Retinal Vessel Parameters in 6 to 12-Year-Old Malay Girls

**DOI:** 10.1371/journal.pone.0170014

**Published:** 2017-01-20

**Authors:** Evelyn Li Min Tai, Ling-Jun Li, Wan Hitam Wan-Hazabbah, Tien-Yin Wong, Ismail Shatriah

**Affiliations:** 1 Department of Ophthalmology, School of Medical Sciences, Health Campus, Universiti Sains Malaysia, Kubang Kerian, Kelantan, Malaysia; 2 Hospital Universiti Sains Malaysia, Kubang Kerian, Kelantan, Malaysia; 3 Singapore Eye Research Institute, Singapore National Eye Centre, Singapore; 4 Duke-NUS Medical School, Singapore; 5 Centre for Eye Research Australia, University of Melbourne, Melbourne, Australia; Tokyo Institute of Technology, JAPAN

## Abstract

**Purpose:**

Retinal vessel analysis is affected by both systemic and ocular factors. Malays are the major ethnicity in South East Asia. Data on the retinal microvasculature in Malays is limited, especially among children. We aim to evaluate the influence of ocular biometry on retinal vessel parameters in young Malay girls.

**Methods:**

This was a cross-sectional, hospital-based study involving 86 Malay girls aged 6 to 12 years old in Hospital Universiti Sains Malaysia from 2015–2016. Ocular examination, refraction, biometry, retinal photography, and anthropometric measurements were performed. The central retinal arteriolar equivalent (CRAE), central retinal venular equivalent (CRVE) and overall fractal dimension (Df) were measured using validated computer-based methods (Singapore I vessel analyzer, SIVA version 3.0, Singapore). The associations of ocular biometry and CRAE, CRVE and Df were analyzed using multivariable analysis.

**Results:**

The mean CRAE, CRVE and Df in Malay girls were 171.40 (14.40) um, 248.02 (16.95) um and 1.42 (0.05) respectively. Each 1 mm increase in axial length was associated with a reduction of 4.25 um in the CRAE (p = 0.03) and a reduction of 0.02 in the Df (p = 0.02), after adjustment for age, blood pressure and body mass index. No association was observed between axial length and CRVE. Anterior chamber depth and corneal curvature had no association with CRAE, CRVE or Df.

**Conclusion:**

Axial length affects retinal vessel measurements. Narrower retinal arterioles and reduced retinal fractal dimension were observed in Malay girls with longer axial lengths.

## Introduction

The human microvasculature, which previously could be evaluated only by relatively invasive methods, is now gradually becoming an open book to us via the retinal circulation [1, 2]. Direct visualization of the retinal vasculature has been simplified by the advent of digital fundus photography, which is rapidly evolving as an integral part of the diagnosis and monitoring of a range of conditions [3]. In adults, retinal vascular calibers and retinal fractal dimensions have been associated not only with ocular problems like diabetic retinopathy [4–6], but also with various systemic diseases, including hypertension [7, 8], coronary heart disease [9, 10] and stroke [11, 12]. Studies in children are comparatively few, and the available data on these parameters in children has focused mainly on Caucasian and East Asian subjects in Sydney [13], with the contribution from our region being mainly Singaporean Chinese children [14, 15].

A significant proportion of the population living in South East Asia fall under the category of ‘Malay’, or ‘Austronesian people’ [16]. This indigenous group of people has been estimated to number approximately 300 million in South East Asia [17]. However, to the best of our knowledge, apart from a multi-ethnic study by Cheung et al in 2007, no other studies of retinal vessel calibers or fractals in children have included this large ethnic group [14, 18]. In children, the general absence of confounding factors related to systemic and ocular disease makes them an ideal study population not only for acquiring normative data, but also in analyzing the relationship between anatomico-physiological factors and retinal vessel parameters [14]. As retinal vessel analysis has been reported to be affected by a variety of factors, which may differ in different populations, we aim to evaluate the influence of ocular biometry on retinal vessel caliber and retinal fractal dimension in young Malay girls.

## Materials and Methods

### Study Population

This was a hospital-based, cross-sectional prospective study involving 86 Malay girls aged 6 to 12 years old, conducted in the Ophthalmology Clinic of Hospital Universiti Sains Malaysia between January 2015 and March 2016. The study was approved by the Human Research Ethics Committee USM (USM/JEPeM/1403119) and the conduct of the study followed the tenets of the declaration of Helsinki.

Subjects who fulfilled the inclusion and exclusion criteria were invited to participate. They were included into the study if they were aged between 6 and 12 years old at the time of examination, had best corrected visual acuity better than 6/12, had normal anterior and posterior segment findings and were Malay (both parents and grandparents were Malay). Exclusion criteria was strabismus, amblyopia, optic nerve abnormalities, high refractive errors (based on spherical equivalent of ±4.0 diopters), history of ocular trauma, ocular pathology, systemic illnesses or developmental delay.

Screening was performed by measuring distance visual acuity monocularly using a Snellen chart for distance (Reichert, NY, USA) at six meters. A comprehensive eye examination included a cover test, assessment of extraocular motility, pupillary examination, anterior segment examination and complete retinal evaluation to rule out any ocular conditions which would have precluded participation in the study. Informed written consent for participation in the study was obtained from at least one parent, and verbal assent from the subject.

### Refraction and Measurement of Ocular Biometric Parameters

Refraction was performed using an autokeratorefractometer (model RK5; Canon, Inc). Three consecutive readings of sphere and cylinder were obtained; a difference between the lowest and highest readings of up to 0.25 diopters was considered acceptable. Spherical equivalent was taken as the value of the sphere plus half of the value of the cylinder. A non-contact partial coherence interferometer (IOL Master, Carl Zeiss Meditec, Inc, Germany) was used to measure the axial length of the right eye. The mean axial length was derived from an average of five consecutive readings, with a signal-to-noise ratio of more than 2 mm, and a difference of up to 0.05 mm between the lowest and highest reading was considered acceptable. Corneal curvature and anterior chamber depth were likewise derived from a mean of five consecutive readings.

### Retinal Photography and Measurement of Retinal Vascular Caliber

After dilation with topical phenylephrine 2.5% and tropicamide 1%, 45 degree digital retinal photographs were taken of the optic disc and macula bilaterally using a digital fundus camera (Model VX-10/KOWA/Japan). Retinal images were then analyzed by a single grader, masked to participant identity and characteristics, using the Singapore I Vessel Algorithm (SIVA) 4.0 cloud software. Right eye fundus photographs were analyzed. In cases where these were ungradable, the images from the left eye were used.

SIVA is a semi-automated computed software, in which all retinal vessels greater than 25 um in diameter located between one-half to two disc diameters from the optic disc margin are outlined, their edges marked using a pixel density histogram, and the retinal arteriolar or venular calibers calculated using the Knudtson-Parr-Hubbard formula [19–21]. This formula summarizes the diameters of the six largest arterioles and venules to generate a central retinal arteriolar equivalent (CRAE) and central retinal venular equivalent (CRVE) respectively.

The Bengtsson formula was used to correct for the effect of ocular magnification on retinal vascular caliber measurement [22, 23]. Fractal dimension was calculated by the software from the previously outlined retinal vessels using the ‘box-counting method’, in which the digital retinal image was compartmentalized into equally-sized squares (i.e. boxes), and the number of squares through which the skeletonized (i.e outlined) segments of retinal vessels pass was counted, after which the process was repeated using a differently-sized square [24, 25]. The fractal dimension was the gradient of the logarithm of the number of squares through which the vessel outline passes against the logarithm of the size of the square [7]. Larger values indicated a more complex branching pattern [26].

### Anthropometric Measurements

Height and weight were measured with the patient standing barefoot, according to a standard protocol of height and weight measuring scale (Seca model 220; Seca, Hamburg, Germany). Height was recorded to the nearest 1 mm while weight was recorded to the nearest 0.1 kg. Body mass index (BMI) was calculated as weight divided by the height squared (kg per meter squared).

Using an appropriate-sized cuff (bladder length approximately 80% and width 40% of the arm circumference), blood pressure and heart rate was measured in the sitting position after 5 minutes of rest, using a digital automated sphygmomanometer. Two separate readings were taken, after which the average systolic and diastolic blood pressure was calculated. A third attempt would be added if the difference between the first two readings were greater than 10 mm Hg in systolic blood pressure (SBP) and/or 5 mm Hg in diastolic blood pressure (DBP). The formula used to obtain mean arterial pressure (MAP) was one-third of the systolic blood pressure plus two-thirds of the diastolic blood pressure. A data collection sheet was used to document all relevant parameters.

### Statistical Analysis

Sample size calculation was performed using G*power 3.1.9.2, with α set at 0.05, power at 0.80 and effect size at 0.15. Statistical analysis was performed using IBM SPSS Statistics for Windows, Version 22.0 (Armonk, NY: IBM Corp). Simple and multiple linear regression were performed to determine the effect of axial length, corneal curvature and anterior chamber depth on retinal vascular calibers and retinal fractal dimension. Two multivariate models were built; Model 1 was adjusted for age, and Model 2 adjusted for age, blood pressure and body mass index. Potential modifiers were examined in stratified analysis. All probabilities quoted are two-sided, and a significant *P* value was defined as <0.05.

## Results

A total of 86 children were included in this study. The mean age was 9.44 ± 1.75 years. Other clinical characteristics; SBP, DBP, MAP and BMI are summarized in Table 1.

**Table 1:** Demographic and Clinical Characteristics.

Characteristics	Mean ± SD
Age, years	9.44 ± 1.75
SBP, mmHg	107.84 ± 11.46
DBP, mmHg	67.50 ± 8.60
MAP, mmHg	80.95 ± 8.47
BMI, kg/m^2^	21.30 ± 4.41

SBP, systolic blood pressure; DBP, diastolic blood pressure; MAP, mean arterial pressure; BMI, body mass index; SD, standard deviation.

The mean spherical equivalent was -0.47 ± 0.72 (diopter). After correcting for ocular magnification, the mean CRAE and CRVE were 171.40 ± 14.40 um and 248.02 ± 16.95 um respectively. The mean retinal fractal dimension was 1.42 ± 0.05. Other ocular parameters are listed in Table 2.

**Table 2:** Ocular Parameters.

Ocular Parameters	Mean ± SD
IOP, mmHg	17.38 ± 2.92
SE, diopter	-0.47 ± 0.72
Axial length, mm	22.91 ± 0.75
Anterior chamber depth, mm	3.41 ± 0.22
Average cornea curvature, mm	43.74 ± 1.56
CRAE, μm	170.07 ± 14.38
CRVE, μm	246.08 ± 16.75
Corrected CRAE, μm	171.40 ± 14.40
Corrected CRVE, μm	248.02 ± 16.95
Total retinal fractal dimension, Df	1.42 ± 0.05

IOP, intraocular pressure; SE, spherical equivalent; CRAE, central retinal arteriolar equivalent; CRVE, central retinal venular equivalent; SD, standard deviation.

Table 3 shows that in models adjusted for age, BMI and MAP, each 1 mm increase in axial length was associated with a reduction of 4.25 um in the CRAE (p = 0.03) and a reduction of 0.016 in the Df (p = 0.024). No association was found between axial length and CRVE. Anterior chamber depth and corneal curvature were not associated with CRAE, CRVE or Df.

**Table 3:** Multiple linear regression in association between ocular biometric parameters, retinal vascular caliber and retinal fractal dimension.

Per SD increase	Corrected CRAE (zone C)			Corrected CRVE (zone C)			Retinal fractal dimension		
	Beta, SE			Beta, SE			Beta, SE		
	Unadjusted	Model 1	Model 2	Unadjusted	Model 1	Model 2	Unadjusted	Model 1	Model 2
Axial length, mm	-5.16, 1.86 (p = 0.007*)	-5.32, 1.89 (p = 0.006*)	-4.25, 1.89 (p = 0.03*)	-3.61, 2.32 (p = 0.12)	-3.69, 2.34 (p = 0.12)	-3.44, 2.41 (p = 0.16)	-0.017, 0.007 (p = 0.018*)	-0.019, 0.007 (p = 0.008*)	-0.016, 0.007 (p = 0.024*)
Corneal curvature, mm	1.38, 0.92 (p = 0.14)	1.38, 0.93 (p = 0.14)	1.09, 0.90 (p = 0.23)	1.21, 1.13 (p = 0.29)	1.22, 1.14 (p = 0.29)	1.11, 1.14 (p = 0.33)	0.003, 0.003 (p = 0.34)	0.003, 0.003 (p = 0.39)	0.002, 0.003 (p = 0.53)
Anterior chamber depth, mm	-15.62, 6.54 (p = 0.019*)	-16.32, 6.68 (p = 0.017*)	-10.54, 6.97 (p = 0.13)	-1.44, 8.19 (p = 0.86)	-1.45, 8.39 (p = 0.86)	-2.46, 8.87 (p = 0.78)	-0.034, 0.025 (p = 0.17)	-0.043, 0.025 (p = 0.09)	-0.031, 0.026 (p = 0.25)

Model 1 adjusted for age. Model 2 adjusted for age, BMI and MAP.

## Discussion

Although Malays, whom the anthropologist Blumenbach described as “tawny-coloured, with black hair”, comprise the main ethnic group in South East Asia, the data on retinal vessel parameters in Malays, especially children, is limited [27, 28]. We present new data of the association of ocular biometry with retinal vascular caliber and retinal fractal dimensions in school-going girls of Malay ethnicity. Table 4 summarizes the literature on this association, including our findings.

**Table 4:** Studies of the association of ocular biometry with retinal vascular caliber.

Study reference	Study, number of gradable images	Ethnicity, n (%)	Ocular Biometric Parameter	Mean age (sd), range	Effect on CRAE (per 1 mm increase in each parameter)	Effect on CRVE (per 1 mm increase in each parameter)
Current study, 2016	86	Malay: 86 (100%)	Axial length	9.44 (1.75) years, 6 to 12 years	β = -4.25 (p = 0.03)	β = -3.44 (p = 0.16)
			Corneal curvature		β = 1.09 (p = 0.23)	β = 1.11 (p = 0.33)
			Anterior chamber depth		β = -10.54 (p = 0.13)	β = -2.46 (p = 0.78)
Gopinath et al,2013 [29]	SPEDS, 5287	Caucasian: 3241 (61.3%); East Asian: 831 (15.7%); Southeast Asian: 272 (5.2%); Middle Eastern: 324 (6.1%); Others: 619 (11.7%)	Axial length	36 to <72 months	β = 3.67 (p = 0.005)	β = -6.53 (p < 0.001)
				6 years old	β = -5.30 (p < 0.001)	β = 7.12 (p = 0.01)
				12 years (n	β = -3.96 (p < 0.001)	β = -6.72 (p < 0.001)
				16–17 years	β = -4.03 (p < 0.001)	β = -6.85 (p < 0.001)
			Corneal curvature	36 to <72 months	β = -14.53 (p < 0.001)	β = -14.29 (p = 0.004)
				6 years old	β = -10.08 (p < 0.001)	β = -14.36 (p < 0.001)
				12 years	β = -9.32 (p < 0.001)	β = -12.92 (p < 0.001)
				16–17 years	β = -6.77 (p < 0.001)	β = -11.84 (p < 0.001)
Li et al, 2011 [30]	STARS, 469	Chinese: 469 (100%)	Axial length	60.68 (7.3) months	β = -3.52 (p = 0.023)	β = -5.55 (p = 0.008)
			Corneal curvature		β = -5.85 (p = 0.093)	β = -13.79 (p = 0.004)
			Anterior chamber depth		β = 4.76 (p = 0.202)	β = 3.55 (p = 0.898)
Cheung et al., 2007 [31]	SCORM, 767	Chinese: 654 (85.3%); Malay: 75 (9.8%); Others: 38 (4.9%)	Axial length	7.8 (0.78) years, 7 to 9 years	β = 0.44 (p = 0.31)	Β = 0.70 (p = 0.25)

SCORM; Singapore Cohort Study of the Risk Factors for Myopia; STARS, Strabismus, Amblyopia, and Refractive Error Study in Singaporean Chinese Pre-schoolers; SPEDS, Sydney Paediatric Eye Disease Study.

We observed that axial length was significantly associated with CRAE. This is consistent with the results of Gopinath et al [29], who evaluated children with no significant refractive error, and Li et al [30], who corrected for ocular magnification. On the contrary, Cheung et al found that after adjusting for ocular magnification, the association between longer axial length and narrower retinal vessels disappeared [31]. As refractive errors are known to affect the measured dimensions of retinal vessels on fundus photography due to differences in ocular magnification, it is important to adjust for this difference. It has been postulated that a longer axial length is associated with a thinner ocular wall and stretched, attenuated retinal vessels [32]. The association of increased axial length with narrowed retinal arterioles may explain the reduced efficiency of the circulation in myopic eyes and indirectly, be related to the pathophysiologic changes seen in this condition [33–36].

We noted that anterior chamber depth and corneal curvature were not associated with either CRAE or CRVE. Among Chinese pre-schoolers, Li et al found no significant effect of anterior chamber depth on retinal vessel calibers [30]. Among studies evaluating corneal curvature, however, the results are conflicting. Li et al examined 469 Chinese preschool children and observed that a larger corneal curvature was significantly associated with a narrower CRVE, but not CRAE [30]. Conversely, Gopinath et al reported in their study comprising approximately 5000 preschool and school-aged children, of which 60% were Caucasian, that this association was true for CRAE as well as CRVE [29]. As statistical significance is affected not only by effect size, but also sample size, differences in the latter between our study and previous research may account for these conflicting results.

The association of longer axial lengths with reduced retinal fractal dimensions observed in our study has never been published previously. Retinal fractal dimension acts as a proxy to represent the geometric pattern of the retinal microvasculature, and reflects the complexity of the branching pattern. As fractal dimension is a ratio, rather than an absolute measurement, it is not affected by the effect of magnification [37]. Retinal fractal dimensions have previously been shown to be affected by blood pressure [15] and nutrition [38], but not by body mass index [39]. Smaller retinal fractal dimensions have been observed in children with higher blood pressure, leading researchers to hypothesize that this change may reflect rarefaction of the microvasculature [15]. At this point in time, we are unable to explain the significance of the observed association between axial length and retinal fractal dimension. One possible explanation for it is that when the rate of eyeball elongation surpasses that of the retinal vessels, the latter become stretched and attenuated, resulting in a reduced fractal dimension. Alternately, axial length may somehow affect the measurement of this retinal vessel parameter by yet unknown mechanisms. Future research is required to explore the clinical relevance of these new findings, as no prior studies have explored this association.

We found that measurement of retinal vessel parameters in children is a simple, convenient and non-invasive method of assessing the body’s microvasculature. Another strength of our study is its homogenous sample of the main ethnic group in South East Asia, thus eliminating any effect of ethnic variations on retinal vessel measurements. Furthermore, use of a standardized protocol for ocular biometry, a validated software for retinal vessel analysis, and adjustment for potential confounders ensures the reliability of these measurements.

Few limitations of this study must be acknowledged. First, all our study participants were girls. Although Cheung et al found no significant gender differences in retinal vascular caliber among children [14], we acknowledge that repeating this study among boys will provide a better overall clinical picture of the effect of axial length in children. As this is a cross-sectional study, further prospective studies would give a better picture of the temporal changes in retinal vessels. Also, the potential for measurement error due to magnification with regard to retinal vessel calibers cannot be fully eliminated, but only minimized. As the formulas developed to adjust for magnification errors still do not encompass the full scope of camera and optical magnification, quantification of absolute retinal vessel diameters remains challenging. As such, retinal fractal dimension may be a better reflection of vessel parameters than retinal vessel caliber.

## Conclusion

Our study shows that axial length significantly affects retinal vessel caliber and fractal dimension in Malay girls. Adjustment for axial length is necessary for future retinal vessel analysis.

## Supporting Information

S1 Fig Sample of SIVA screen. (JPG)

S1 Table Systemic, ocular and retinal vascular parameters of study subjects. (PDF)
