# Supplementary figures and images for: Effect of Axial Eye Length on Retinal Vessel Parameters in 6 to 12-Year-Old Malay Girls
Source: PLoS One. 2017 Jan 20;12(1):e0170014. doi: 10.1371/journal.pone.0170014 (PMC5249240; doi:10.1371/journal.pone.0170014)

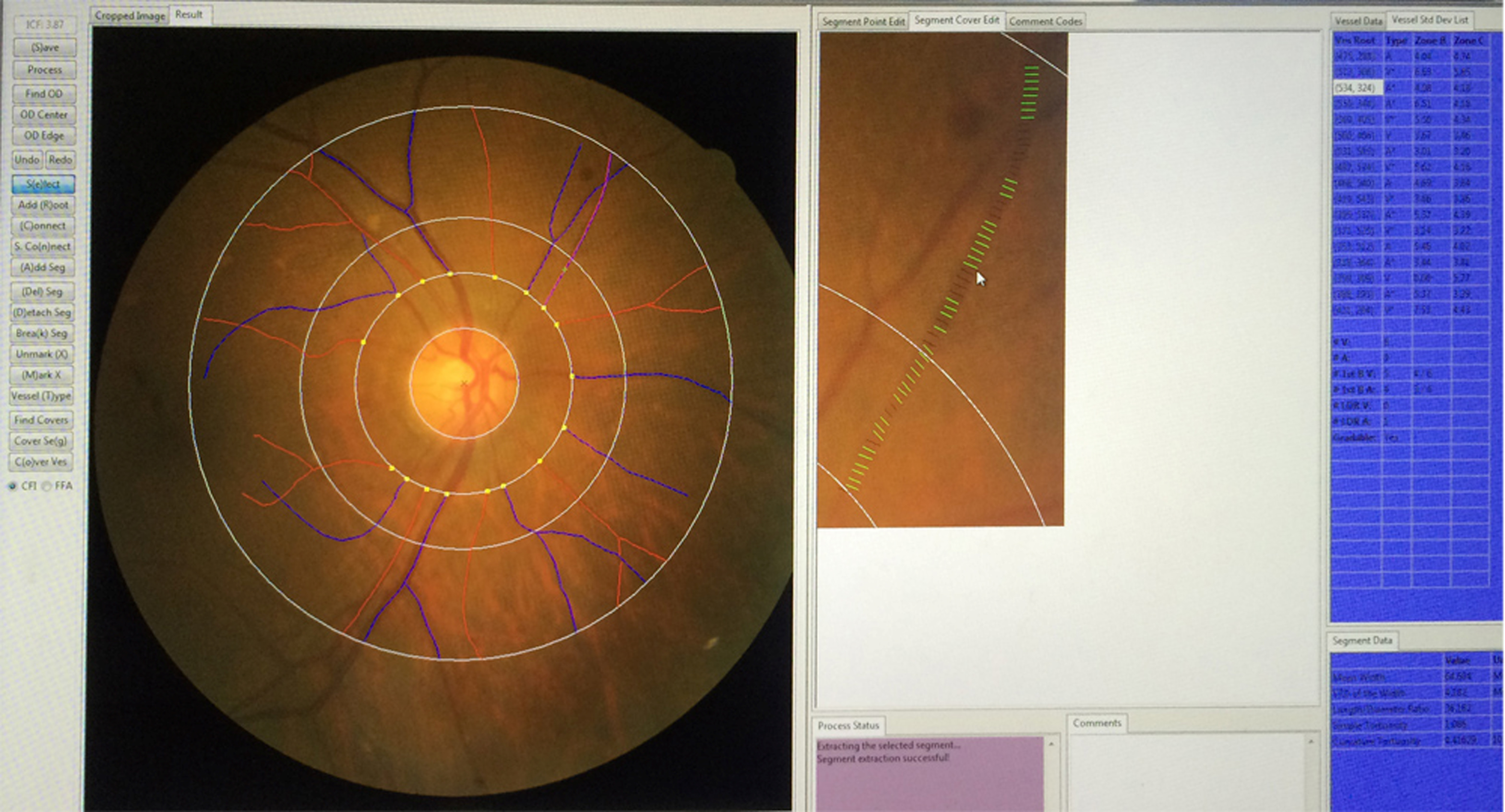

Supplement: S1 Fig — (JPG) [file pone.0170014.s001.jpg]
