# Supplementary material for: Effect of Axial Eye Length on Retinal Vessel Parameters in 6 to 12-Year-Old Malay Girls
Source: PLoS One. 2017 Jan 20;12(1):e0170014. doi: 10.1371/journal.pone.0170014 (PMC5249240; doi:10.1371/journal.pone.0170014)
Supplement: S1 Table — (PDF) [file pone.0170014.s002.pdf]

S1 Table. Systemic, ocular and retinal vascular parameters of study subjects.

Number	Body mass index	Mean arterial pressure	Axial length	Average corneal curvature	Anterior chamber depth	Corrected central retinal arteriolar equivalent	Corrected central retinal venular equivalent	Fractal dimension
1	21.40	92.33	22.32	44.27	3.45	157.192	241.843	1.370
2	22.51	80.00	23.35	42.23	3.33	161.267	244.500	1.438
3	25.68	92.33	23.34	43.80	3.38	166.116	253.397	1.452
4	16.91	79.67	22.55	43.39	3.17	185.126	227.591	1.346
5	24.88	93.33	24.68	41.93	3.75	160.742	237.475	1.471
6	17.26	80.00	22.63	44.51	3.62	171.291	254.725	1.461
7	17.23	69.67	22.02	43.36	3.09	191.760	303.809	1.402
8	25.57	90.33	21.68	46.08	3.14	175.703	285.306	1.480
9	21.36	71.33	22.28	44.68	3.19	185.173	261.278	1.450
10	14.12	73.00	21.28	47.61	3.36	169.307	248.576	1.461
11	22.05	69.67	21.37	47.38	3.61	200.686	243.671	1.508
12	15.59	80.33	23.29	44.47	3.56	152.587	231.644	1.444
13	13.77	78.00	23.52	42.06	3.24	155.538	252.143	1.426
14	16.74	91.33	21.78	46.63	3.41	179.832	251.444	1.411
15	28.13	94.00	23.49	43.14	3.66	155.307	253.666	1.452
16	26.11	90.00	22.84	44.60	3.77	176.909	289.133	1.410
17	21.89	79.33	23.78	42.70	3.25	161.030	221.681	1.423
18	31.69	96.00	23.21	43.33	3.64	166.633	270.709	1.447
19	14.12	60.33	22.02	44.65	3.26	178.997	250.790	1.476
20	16.44	83.67	22.20	44.48	3.23	167.394	230.484	1.464
21	16.64	89.33	24.29	40.70	3.11	154.701	224.495	1.436
22	15.70	73.67	22.37	44.33	3.04	164.972	237.887	1.393
23	13.89	78.00	22.24	45.03	3.08	183.484	259.351	1.402
24	16.35	75.33	22.70	42.84	3.33	170.989	245.916	1.356
25	11.72	75.67	22.78	43.63	3.46	180.316	251.404	1.391
26	20.79	83.67	23.69	41.63	3.61	162.253	265.875	1.440
27	16.28	75.67	23.23	43.00	3.42	192.616	258.330	1.354

28	12.71	90.33	23.25	43.89	3.63	157.542	228.277	1.408
29	18.38	82.00	23.49	43.30	3.46	196.655	269.179	1.391
30	21.23	85.00	23.53	42.25	3.75	167.933	245.327	1.353
31	18.75	87.00	23.10	45.07	3.40	141.436	251.523	1.308
32	21.12	72.33	22.92	42.49	3.34	188.772	285.010	1.450
33	16.71	80.67	24.12	44.05	3.99	188.362	279.908	1.318
34	30.90	92.33	21.56	46.91	3.58	193.513	283.720	1.468
35	23.96	87.00	23.11	43.62	3.48	172.537	265.088	1.473
36	24.15	89.67	23.33	42.33	3.20	169.998	260.174	1.414
37	20.98	82.00	23.06	42.25	3.42	155.590	219.406	1.472
38	20.16	68.00	21.78	46.97	3.68	180.803	254.509	1.452
39	18.81	74.00	23.35	42.68	3.45	195.278	245.467	1.414
40	19.51	94.33	23.54	41.75	3.10	186.269	246.510	1.426
41	20.62	84.33	22.54	43.67	3.05	175.436	262.641	1.429
42	19.79	69.33	22.27	44.28	3.37	182.453	260.421	1.409
43	22.05	97.67	23.75	42.87	3.42	167.866	238.904	1.293
44	24.47	86.00	22.78	45.21	3.68	153.985	265.891	1.398
45	21.72	77.67	24.05	41.02	3.24	169.065	270.606	1.407
46	25.02	77.67	23.84	43.90	3.75	147.575	251.915	1.399
47	34.22	86.67	23.66	41.95	3.65	145.144	217.862	1.228
48	24.89	86.33	22.38	45.54	3.59	165.921	240.942	1.449
49	24.14	72.67	23.94	42.44	3.20	168.852	244.500	1.408
50	22.37	73.00	22.57	45.62	3.38	177.706	250.409	1.368
51	24.14	81.67	23.53	44.13	3.25	162.268	265.308	1.408
52	19.75	80.67	23.55	44.35	3.35	165.816	242.361	1.445
53	20.50	82.33	23.87	45.75	3.65	179.474	248.950	1.369
54	23.28	82.33	22.52	43.75	3.75	162.477	257.970	1.379
55	22.05	88.67	23.85	44.13	3.65	160.619	248.508	1.459
56	25.80	94.67	22.50	40.35	3.75	163.054	255.544	1.417
57	20.17	77.00	24.50	43.00	3.25	168.374	250.310	1.436
58	17.75	79.67	23.16	42.30	3.49	181.904	267.894	1.453
59	28.67	93.33	22.45	45.46	3.50	185.322	259.390	1.412

60	25.43	77.00	23.44	43.10	3.63	170.713	246.425	1.396
61	23.28	84.67	24.33	40.20	3.36	160.620	241.990	1.415
62	25.08	75.33	22.56	43.50	3.16	183.271	258.017	1.480
63	22.68	79.00	21.89	46.30	3.10	197.513	263.193	1.420
64	21.21	88.67	22.84	44.30	3.51	187.135	264.771	1.388
65	29.44	90.67	22.89	45.20	3.75	147.975	257.609	1.403
66	24.14	77.00	22.60	45.68	3.29	151.878	255.333	1.483
67	19.98	76.33	23.32	41.85	3.30	180.192	265.133	1.521
68	25.73	79.00	22.81	43.64	3.58	161.695	247.587	1.405
69	21.91	74.00	23.12	42.22	3.25	177.791	257.096	1.501
70	22.77	79.00	22.62	44.32	3.44	167.540	245.833	1.401
71	14.79	72.33	21.72	43.65	3.37	183.719	274.919	1.448
72	16.45	76.00	22.11	44.15	3.24	165.337	245.928	1.481
73	22.77	89.00	22.62	44.35	3.44	169.369	242.680	1.395
74	17.85	68.00	22.39	43.44	3.27	151.048	232.472	1.402
75	19.17	66.00	22.95	44.39	2.99	185.453	266.544	1.336
76	24.31	71.67	21.90	43.61	2.93	189.353	257.323	1.438
77	21.81	59.33	22.18	44.51	3.56	166.833	252.534	1.490
78	20.01	83.33	22.01	44.41	3.29	157.784	242.993	1.447
79	18.86	81.33	23.07	41.77	3.23	175.462	244.071	1.422
80	22.11	74.00	22.42	43.27	3.27	175.934	273.148	1.471
81	22.68	74.67	22.16	45.24	3.54	151.989	221.199	1.404
82	29.59	89.00	23.45	43.24	3.42	172.050	273.690	1.425
83	18.14	92.67	22.86	42.68	3.39	161.610	231.645	1.381
84	24.89	89.00	22.38	45.54	3.59	168.638	235.719	1.419
85	21.91	74.00	23.12	42.22	3.25	178.339	258.577	1.496
86	21.72	68.33	24.00	41.11	3.21	184.326	254.468	1.381
